# Age-range–matched external validation of standard liver volume equations: Methodological re-evaluation of 13 regression models

**DOI:** 10.1371/journal.pone.0351983

**Published:** 2026-07-08

**Authors:** Hiroshi Imamura, Ryota Ito, Hirofumi Ichida, Ryuji Yoshioka, Yoshihiro Mise, Shuko Nojiri, Katsuhiro Sano, Atsuyuki Yamataka, Akio Saiura

**Affiliations:** 1 Department of Hepatobiliary-Pancreatic Surgery, Juntendo University Graduate School of Medicine, Tokyo, Japan; 2 Medical Technology Innovation Centre, Juntendo University Graduate School of Medicine, Tokyo, Japan; 3 Department of Radiology, Juntendo University Graduate School of Medicine, Tokyo, Japan; 4 Department of Pediatric General and Urogenital Surgery, Juntendo University Graduate School of Medicine, Tokyo, Japan; University of Tsukuba, JAPAN

## Abstract

**Background and aim:**

Accurate estimation of standard liver volume (SLV) is essential in hepatobiliary surgery, particularly in living donor liver transplantation and hepatic resection. Numerous and substantially divergent SLV equations have been proposed, yet it remains unclear which are methodologically valid. Although several comparative and validation studies have been reported, these have typically evaluated equations beyond their original derivation ranges, thereby conflating external validity with extrapolation and ignoring physiologically distinct processes across the lifespan. As a result, a proper external validation of existing SLV equations has not been systematically performed.

**Methods:**

Thirteen previously proposed SLV formulae were selected for methodologically constrained external validation in individuals without known liver disease (n = 1152). Each equation was evaluated strictly within its original derivation age range using age-range–matched cohorts. Agreement between estimated SLV and computed tomography–measured total liver volume (TLV) was assessed using Bland–Altman analysis and intraclass correlation coefficients (ICC). Model performance was further examined in older adults and across subgroups defined by sex, obesity, and emaciation.

**Results:**

Urata’s (proportional function of body surface area) and Noda’s (power function of body weight) formulae showed the highest agreement within their derivation age ranges. When extrapolated beyond these ranges, systematic age-dependent bias emerged, consistent with age-related hepatic involution rather than model structure. TLV remained stable between 20 and 49 years of age but declined linearly after 50 years at a rate of 2.7–2.8% per five years. Obesity was associated with increased TLV due to steatosis, whereas sex and emaciation had minimal effects.

**Conclusions:**

This study demonstrates that failure to respect derivation age ranges leads to misleading assessments of SLV equation performance. Age-range–matched external validation clarifies prior inconsistencies and provides a generalizable framework for evaluating regression-based prediction models, with implications for both clinical epidemiology and quantitative medical research.

## Introduction

The human liver possesses a physiologically “appropriate” volume for each individual, determined by the metabolic demands of the whole body. In partial liver transplantation (i.e., living-donor and split-liver transplantation), the graft is often smaller than this native requirement. Therefore, accurate estimation of graft volume and its proportion relative to a person’s normal total liver volume (TLV) is crucial, as undersized grafts can jeopardize recipient outcomes. Although graft volume can be measured directly using computed tomography (CT) images of the donor’s liver, measurement of the recipient’s liver volume cannot serve as a reference for what the liver *should* be, because the recipient’s liver is often pathologically hypertrophic or atrophic.

To address this problem, Urata et al. (1995) first introduced the concept of standard liver volume (SLV), defined as an ideal liver volume based on an almost proportional relationship between body surface area (BSA) and TLV in healthy individuals aged 0–27 years [1]. Since then, numerous SLV formulae have been proposed using anthropometric parameters such as BSA and body weight (BW), as summarized in Table 1 [1–17]. Yet these equations diverge substantially, and previous validation studies have produced inconsistent conclusions [10,18], a situation that has created what may be described as a “Tower of Babel” in the SLV literature.

**Table 1:** Seventeen formulae calculating standard liver volume previously reported.

Study	Year	Formulae	Country	*n*	Age, y^a^	Range	Modality
Deland^2^	1968	1020 × BSA −220	USA	625	N/A	N/A	Autopsy
**Urata** ^**1**^	1995	706.2 × BSA + 2.4	Japan	96	11.1	<1-27	CT
**Noda** ^**3**^	1997	49.54 × BW^0.78^	Japan	54	N/A	<1-22	CT
**Lin** ^**4**^	1998	13 × HT + 12 × BW −1530	Taiwan	33	50	25-67	CT
Heinemann^5^	1999	1072.8 × BSA −345.7	Germany	1332	50.6	N/A	Autopsy
**Vauthey** ^**6**^	2002	1267.28 × BSA – 794.41	USA, Switzerland, Belgium	292	54	14-90	CT
**Yoshizumi** ^**7**^	2003	772 × BSA	USA	1413	41.9	<1-87	Cadaveric donor graft
**Yu** ^**8**^	2004	21.585 × BW^0.732^ × HT^0.225^	Korea	652	42.4	<1-90	Autopsy
**Choukèr** ^**9**^	2004	16.434 × BW + 11.85 × age – 166 × (sex factor)+452	Germany	728	46.5	16-70	Autopsy
**Johnson** ^**10**^	2005	0.722 × BSA^1.176^	UK	5036	N/A	<1-73.7	CT
**Hashimoto** ^**11**^	2006	961.3 × BSA – 404.8	Japan	301	N/A	17-66	CT
**Chan** ^**12**^	2006	12.29 × BW + 50.74 x (sex factor)+218.32	China	159	35.8	16-57	CT
**Yuan** ^**13**^	2008	949.7 × BSA – 48.3 × (age factor) −247.4	China	112	48.7	19-73	CT
**Fu-Gui** ^**14**^	2009	11.508 × BW + 334.024	China	115	36	19-57	CT
Poovathumkadavil^15^	2010	12.26 × BW + 555.65	Saudi Arabia	351	49.2	N/A	CT
Kokudo^16^	2017	58.7 × thoracic width – 3.61 × age – 463.7 × (Race factor) + 203.3	Japan	340	N/A	19-90	CT
**Yang** ^**17**^	2021	(BW < 20 kg) 707 × BSA^1.09^ (BW > 20 kg) Male, 691.9 × BSA^1.09^; Female, 663 × BSA^1.04^	China	792	6.5	<1-18	CT

^a^ mean or median value.

*Note.* Formulae shown in bold were selected for validation in the present study. Selection was based on the availability of a defined derivation age range.

Abbreviations: N/A, not available; BSA, body surface area; BW, body weight; CT, computed tomography; USA, United States of America; UK, United Kingdom.

This inconsistency can be attributed to physiological and methodological issues.

1) Allometric scaling

Allometric scaling describes the relationship between the size of a body part and overall body size and holds universally across biological systems, from insects and reptiles to birds and mammals, and from shrews to blue whales [19,20]. In this context, like most organs, the liver exhibits subproportional (hypoallometric) growth, meaning that it increases in size less than proportionally relative to body mass [20]. This relationship also holds among adult individuals with varying body weights. By contrast, many prior SLV studies implicitly assumed linearity between BW and TLV, thereby failing to account for this fundamental scaling behavior. [4,9,12,14,15].

2) Extrapolation beyond derivation ranges

A key consideration in the use of regression-derived formulae is the scope of their applicability: such equations are valid only within the data range used for their development. Age, in particular, plays a significant role in liver volume. The liver’s proportion of BW decreases from approximately 4% in neonates to 2% in adults during growth [21,22], and further decreases by up to 20% in octogenarians during aging [23,24]. Nevertheless, many prior discussions asserting the superiority of specific formulae compared their performance by extrapolating beyond the original derivation age ranges [6,18]. Such extrapolation distorts slope and intercept behavior, leading, for example, to biologically implausible non-zero intercepts in several adult-derived linear equations [4,6,9,11–14].

3) Age-related reduction in liver volume

Liver volume has been suggested to decrease with aging, based largely on indirect and low-resolution measurements, such as ultrasonography [23,24]. However, no classical SLV equation incorporates age-related change, despite its potential impact on liver-volume estimation in older adults.

Taken together, these issues have prevented the SLV literature from establishing a unified, physiologically grounded framework. To address this gap, we conducted the first age-range–matched, physiology-based external validation of 13 widely used SLV equations in a cohort of 1152 individuals without known liver disease.

## Subjects and methods

This retrospective study was approved by the institutional review board (Approval number: E21-0319). The ethics approval document is attached in the Supporting information file (Ethics approval document). The Informed consent was waived because of the retrospective design. The data for this investigation were accessed for research purpose in 03/10/2025.

### External validation of previously proposed SLV formulae within their original age ranges

Table 1 displays various formulae for calculating SLV, with 13 selected for validation (indicated in bold [1,3,4,6–14,17]). Excluded were three without specified age ranges [2,5,15] and one estimating SLV as a function of thoracic width [16]. The validation cohort comprises all living-donor liver transplantation (LDLT) donor candidates between February 2003 and March 2018 and consecutive patients who underwent elective laparoscopic cholecystectomy (LC) for benign gallbladder disease and received preoperative contrast-enhanced CT examination between February 2009 and March 2022 at the Department of Hepatobiliary-Pancreatic Surgery, and consecutive patients who received contrast-enhanced CT examination for conditions unrelated to hepatobiliary or malignant disease at the Department of Pediatric General and Urogenital Surgery between February 2009 and March 2022, both in Juntendo University Hospital. Individuals with known chronic liver disease, malignancy, or structural liver abnormalities were excluded. Living donor candidates were considered physiologically normal based on comprehensive clinical evaluation, including laboratory testing and imaging. For non-donor subjects, we adopted a consecutive inclusion strategy to minimize selection bias, accepting a trade-off between strict physiological normality and generalizability.

BSA was calculated based on BW and body height (BH) using the Du Bois equation for individuals with BW ≥ 15 kg [25] and the Haycock equation for those with BW < 15 kg [26]. TLV was measured using contrast-enhanced CT images with 1-mm slice thickness and three-dimensional image analysis software (Synapse VINCENT; Fujifilm Corporation, Tokyo, Japan). Liver segmentation was performed using a semi-automated approach, in which hepatic contours and intrahepatic vascular structures were initially detected automatically and subsequently reviewed and, if necessary, manually adjusted by experienced operators. Major intrahepatic vessels were excluded from volume calculation, and focal hepatic lesions, all of which were benign in this cohort, were also excluded. Although the software allows detailed segmental volumetry, the present study focused exclusively on TLV, and segmental volume measurements were not used in the analysis.

SLVs were calculated using the applicable formula for each subject within the age range of the original derivation cohort (estimated SLV). For formulae estimating liver weight rather than volume, liver density was assumed to be 1.07 g/mL to convert values to SLV [7,9].

Agreement between estimated SLV and measured TLV was assessed using modified Bland–Altman plots [27]. In these plots, TLV was plotted on the x-axis and the relative difference (SLV − TLV)/TLV on the y-axis, with TLV treated as the reference standard. Mean bias and limits of agreement (LOA; 95% C.I.) were calculated, and agreement was further quantified using the intraclass correlation coefficient (ICC) [28].

### Verification of SLV formulae in subjects outside the original age ranges and effects of aging, sex, and obesity/emaciation on SLV estimation

The most accurate formulae were selected based on ICC values and examined for their applicability beyond their originally intended age ranges, that is, whether the equation can be extrapolated outside the original age range as it is or whether a modification term is required. We also investigated whether the estimation of SLV maintained accuracy in older individuals for the formulae whose original age range of data extended to 90 years [6–8].

Additionally, we examined whether sex and/or obesity/emaciation affected the relationships between BSA/BW and TLV. We also investigated whether fatty liver was more prevalent among obese individuals and whether fatty liver status influenced liver volume. The presence of fatty liver was determined through mean attenuation measurements in Couinaud segments V–VIII, as described by Pickhardt et al. [29], which detects moderate to severe hepatic steatosis (≥30% at histology) with 53.5% sensitivity and 100% specificity.

### Statistical analyses

Continuous variables were compared using the Wilcoxon test, and categorical data were analyzed with Fisher’s exact test. The modified Bland-Altman plot assessed the agreement between estimated SLV and measured TLV [27]. ICC estimates with 95% C.I. were calculated using a mean-rating (*k* = 2), absolute agreement, 2-way mixed-effects mode [28]. Interpretation was as follows: < 0.5, poor; between 0.50 and 0.75, fair, between 0.75 and 0.90, good; above 0.90, excellent. Multiple group comparisons employed the Dunnett test. Polynomial equations were selected for optimal fitting based on the Akaike Information Criteria (AIC) [30]. Statistical significance was set at *P* < 0.05. All analyses were conducted using SAS (SAS Institute, Cary, North Carolina, USA).

## Results

### External validation of SLV formulae within their original age ranges

The base validation population comprised 1152 subjects: 85 donors (age 19–64 years), 774 patients undergoing LC (age 21–92 years), and 293 other patients (age 0 [16 days] – 29 years) (Table 2).

**Table 2:** Background characteristics of subjects or patients.

	Base population cohort (*n* = 1152)		
	Normal subjects (*n* = 1044)	Obese subject ≥18 years-old (*n* = 28)	Emaciated subjects ≥18 years-old (*n* = 49)
Age, y	51 (16day-92)	50 (22-80)	53 (22-83)
Sex (male: female)	521: 523	18: 10	13: 36
Intention of CT	79 donors 711 elective LC 196 acute abdomen 24 urologic disease 13 gonadal disease 4 trauma 17 others	26 elective LC 2 acute abdomen	6 donors 37 elective LC 6 acute abdomen
BSA (m^2^)	1.60 (0.19-2.27)	1.94 (1.56-2.37)	1.42 (1.18-1.67)
BW(kg)	59 (3-99)	88.9 (65.5-133)	44 (33.4-56)
LV (mL)	726 (397-1237)	1738 (1084-3261)	988 (682-1528)

Note: Subcategory of base population cohort does not include 13 obese and 18 emaciated pediatric subjects.

Data are presented as median (minimum-maximum).

The effect of ageing on the Liver volume was examined in 826 subjects ≥ 20 years old, while that of sex was investigated in 489 subjects ranging from 0 to 49 years old, both selected from a pool of 1044 normal subjects. The effect of obesity/emaciation on liver volume was analysed in all 913 adult (≥18 years old) subjects in the base population cohort.

Abbreviations: CT, computed tomography; LC, laparoscopic cholecystectomy; BSA, body surface area; BW, body weight; TLV, total liver volume.

**Fig 1:**
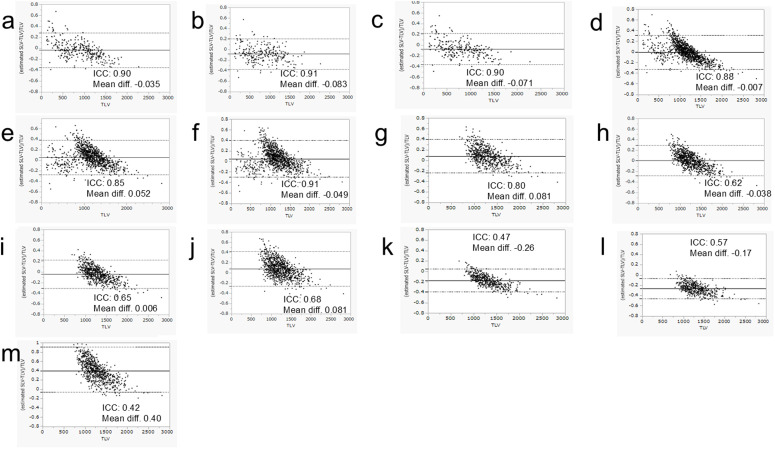
Modified Bland–Altman plots comparing estimated SLV with computed TLV across 13 previously proposed equations. Panels are arranged according to overall agreement based on Bland–Altman assessment, from best-performing to poorest-performing equations.(a) Urata, (b) Noda, (c) Yang, (d) Yoshizumi, (e) Johnson, (f) Yu, (g) Lin, (h) Yuan, (i) Hashimoto, (j) Vauthey, (k) Fu-Gui, (l) Chan, and (m) Choukèr. The x-axis represents TLV (reference standard), and the y-axis represents relative difference (SLV − TLV)/TLV. Solid lines indicate mean bias (fixed bias), and dotted lines indicate LOA. ICC are displayed in each panel; complete parameters including 95% confidence intervals and proportional bias estimates are provided in Table 3. The apparent low proportional bias observed in some equations, particularly Yoshizumi’s, Johnson’s, and Yu’s formulae, should be interpreted cautiously, as visually distinct clustering patterns contributed to attenuation of the apparent overall proportional bias estimates. Abbreviations: SLV, standard liver volume; TLV, total liver volume; ICC, intraclass correlation coefficient. LOA, limit of agreement.

Bland–Altman analysis identified Urata’s, Noda’s, and Yang’s equations as demonstrating the best overall agreement, characterized by mean differences close to zero, minimal outliers, low proportional bias, and high ICC values (Fig 1a–1c). The formulae by Yoshizumi, Johnson, and Yu initially appeared to demonstrate small proportional bias based on overall slope estimates (Fig 1d–1f). However, their plots revealed two distinct clusters across the TLV range, resulting in deceptively small overall slopes. When proportional bias was recalculated within the higher-volume subgroup (TLV > 750 mL), substantial bias became evident for Johnson’s and Yu’s equations (−3.30 × 10 ⁻ ⁴ and −3.53 × 10 ⁻ ⁴, respectively), and a similar clustering-driven distortion was observed for Yoshizumi’s equation. Notably, within the TLV range of 700–1200 mL, representing most adults in routine clinical practice, fixed bias remained pronounced for these equations.

**Table 3:** Parameters of modified Bland-Altman plot and intraclass correlation coefficient for 13 formulae for SLV.

Study	Bland-Altman plot				ICC	
	mean bias	LOA	Proportional bias (x 10^−4^)	outlier (%)	Mean	95%CI
Urata^1^	−0.035	−0.35 - 0.28	−2.28	5.8	0.90	0.83 - 0.94
Noda^3^	−0.083	−0.38 - 0.21	−0.91	5.3	0.91	0.79 - 0.95
Lin^4^	0.081	−0.26 - −0.40	−3.17	1.6	0.80	0.72 - 0.85
Vauthey^6^	0.081	−0.26 - 0.42	−3.26	5.0	0.68	0.59 - 0.74
Yoshizumi^7^	−0.007	−0.33 - 0.32	−2.93	1.2	0.88	0.86 - 0.90
Yu^8^	0.049	−0.30 −0.40	−1.40	1.5	0.91	0.90 - 0.92
Choukér^9^	0.400	−0.059 −0.85	−5.46	0.55	0.42	0.00 - 0.74
Johnson^10^	0.052	−0.27 - 0.38	−1.51	5.1	0.85	0.82 - 0.87
Hashimoto^11^	−0.038	−0.31 - 0.23	−3.19	5.8	0.62	0.51 - 0.70
Chan^12^	−0.26	−0.46 - −0.065	−0.19	1.3	0.47	0.00 - 0.77
Yuan^13^	0.006	−0.28 - 0.29	−3.58	5.0	0.65	0.63 - 0.69
Fu-Gui^14^	−0.17	−0.40 - 0.051	−2.79	1.3	0.57	0.00 - 0.80
Yang^17^	−0.071	−0.36 −0.22	−1.82	7.0	0.90	0.77 - 0.95

Abbreviations: ICC, intraclass correlation coefficient; LOA limit of agreement; C.I., confidence interval.

Lin’s, Yuan’s, Hashimoto’s, and Vauthey’s equations demonstrated moderate agreement with noticeable fixed and/or proportional biases (Fig 1g–1j). In contrast, Fu-Gui’s, Chan’s, and Choukèr’s equations showed substantial fixed bias and wide limits of agreement, indicating poor agreement with measured TLV (Fig 1k–1m).

Correspondingly, ICC values exceeded 0.90 for Urata’s, Noda’s, and Yang’s formulae, ranged between 0.75 and 0.90 for several moderately performing equations, and were below 0.50 for Chan’s and Choukèr’s formulae (Table 3).

Overall, Urata’s, Noda’s, and Yang’s formulae, which predict measured TLV by BSA or BW, were identified as potential candidates for the further examination; however, Yang’s formula was specifically designed to represent pediatric SLV for hepatoblastoma surgery and was developed based on subjects with a mean age of 6.5 years. Moreover, this formula comprises three different equations based on BW and sex, lacking simplicity.

Consequently, Urata’s and Noda’s formulae, which estimate SLVs as a linear function of BSA and a power function of BW, respectively, were selected for further investigation.

### Applicability of Urata’s and Noda’s formulae beyond original age range, and effects of sex and obesity/emaciation

In these analyses, we divided the validation cohort into three groups as follows: subjects with normal weight (*n* = 1044), subjects with obesity (*n* = 41; 28 subjects ≥18 years old, 13 subjects <18 years old), and subjects with emaciation (*n* = 67; 49 subjects ≥18 years old, 18 subjects <18 years old) (Table 2). Obesity was defined as a body mass index (BMI) ≥30 kg/m^2^ in subjects ≥18 years old and ≥90th percentile of the BMI-standard deviation score (SDS) score for those <18 years old. Emaciation was defined as a BMI < 18 kg/m^2^ in subjects ≥18 years old and <10th percentile of the BMI-SDS in those <18 years old [31].

### Effect of aging on SLV estimation

**Fig 2:**
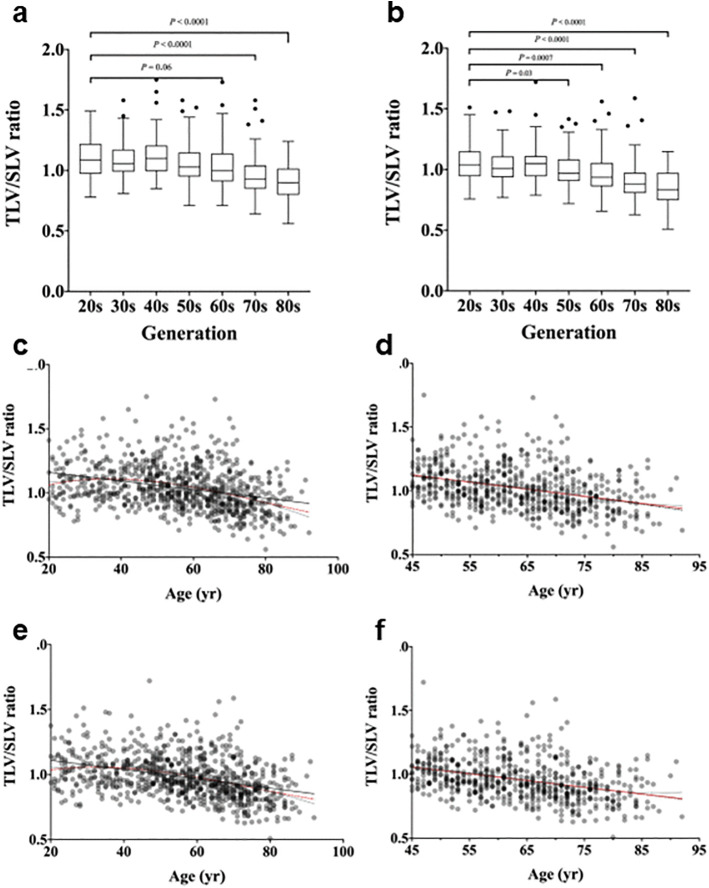
Effect of aging on liver volume in adults. (a, b) TLV/SLV ratios across decades using Urata’s and Noda’s formulae, respectively; data shown as box-and-whisker plots. (c–f) Polynomial regression of TLV/SLV ratio vs. age: (c, d) Urata’s formula; (e, f) Noda’s formula; c and e: all subjects ≥20 years; d and f: subjects ≥45 years. Solid, dashed, and dotted lines indicate linear, quadratic, and cubic fits, respectively. Abbreviations: TLV, total liver volume; SLV, standard liver volume; SD, standard deviation.

This was analyzed in 826 individuals aged ≥20 years, selected from 1044 normal-weight subjects to remove confounding effects of growth. SLV estimation errors were assessed using TLV/SLV ratios by decade (Fig 2a and 2b). Compared to subjects in their 20s, the TLV/SLV ratios by decades declined steadily aged 50 years and older, starting in 50s for Noda’s formula and in the 70s for Urata’s formula.

To analyze this trend more precisely, we plotted the TLV/SLV ratio against age and fitted polynomial curves (Fig 2c and 2d). AIC selected a quadratic model as optimal, indicating a stable ratio until the late 40s followed by a progressive decline. To determine whether this decline was linear or accelerated, we repeated the analysis in subjects aged ≥45 years. AIC supported a linear fit (Fig 2e and 2f), indicating a decline of approximately 2.7–2.8% per five years for both Urata’s and Noda’s equations. Collectively, the TLV/SLV ratio remains constant until the end of the fifth decade and decreases linearly from age 50 onward.

We also tested whether three formulae originally developed in broader age ranges (up to age 90) overestimated SLV in older adults. Indeed, TLV/SLV ratios decreased significantly from the 60s onward for all three (Table 4).

**Table 4:** Age-dependent changes in TLV/SLV ratios for SLV formulae derived from broad age ranges.

Formula	Age group	TLV/SLV ratio, median (IQR)	P value vs. 20s
Vauthey^6^	20s	0.99 (0.88–1.10)	Reference
	60s	0.92 (0.84–1.02)	.023
	70s	0.87 (0.77–0.98)	<.0001
	80s	0.82 (0.76–0.98)	<.0001
Yoshizumi^7^	20s	1.06 (0.96–1.19)	Reference
	60s	0.98 (0.90–1.13)	.078
	70s	0.92 (0.84–1.02)	<.0001
	80s	0.88 (0.79–0.98)	<.0001
Yu^8^	20s	0.99 (0.91–1.10)	Reference
	60s	0.91 (0.84–1.01)	.0027
	70s	0.85 (0.78–0.95)	<.0001
	80s	0.80 (0.73–0.93)	<.0001

Data are presented as median (interquartile range).

*P* values represent comparisons with subjects in their 20s.

TLV, total liver volume; SLV, standard liver volume, IQR, interquartile range.

### Effect of sex on SLV estimation

**Fig 3:**
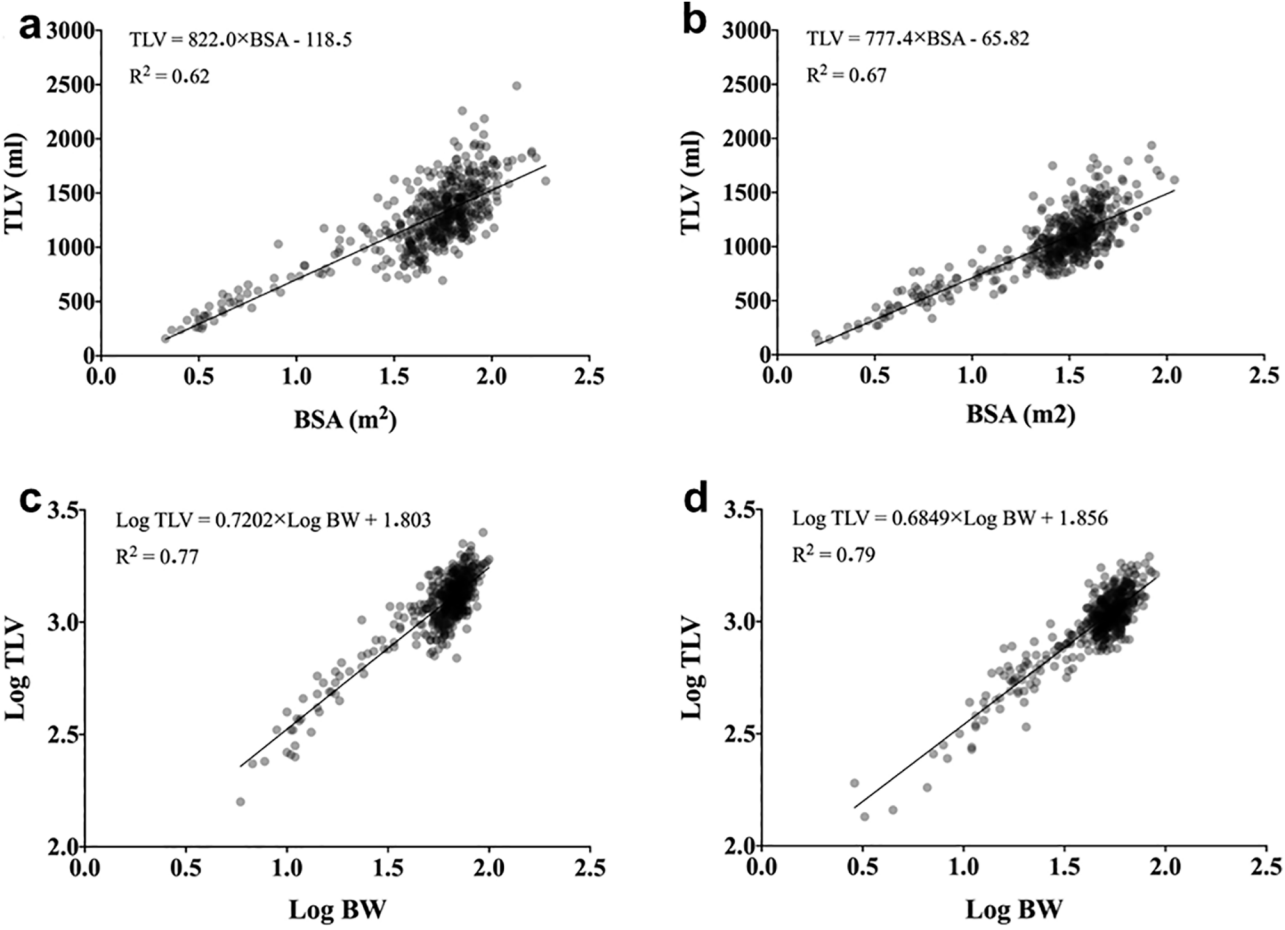
Sex-based comparison of relationship between body mass and liver volume in individuals aged <50 years. (a, b) BSA vs. TLV in males and females, respectively. (c, d) Log-transformed BW vs. TLV in males and females. Abbreviations: BSA, body surface area; BW, body weight; TLV, total liver volume.

This was investigated in 489 individuals (229 males and 260 females) ranging in age from 0 to 49 years, selected from a pool of 1044 normal-weight subjects. To prevent potential interaction resulting from the age-related decrease in liver volume, subjects aged 50 years or older were excluded. BSA–TLV and log BW–log TLV relationships were compared between sexes (Fig 3a-3d). No significant differences were observed in slopes (BSA–TLV: *P* = 0.181; log BW–TLV: *P* = 0.078) or intercepts (*P* = 0.746 and *P* = 0.179, respectively).

### Effect of obesity/emaciation on SLV estimation

**Fig 4:**
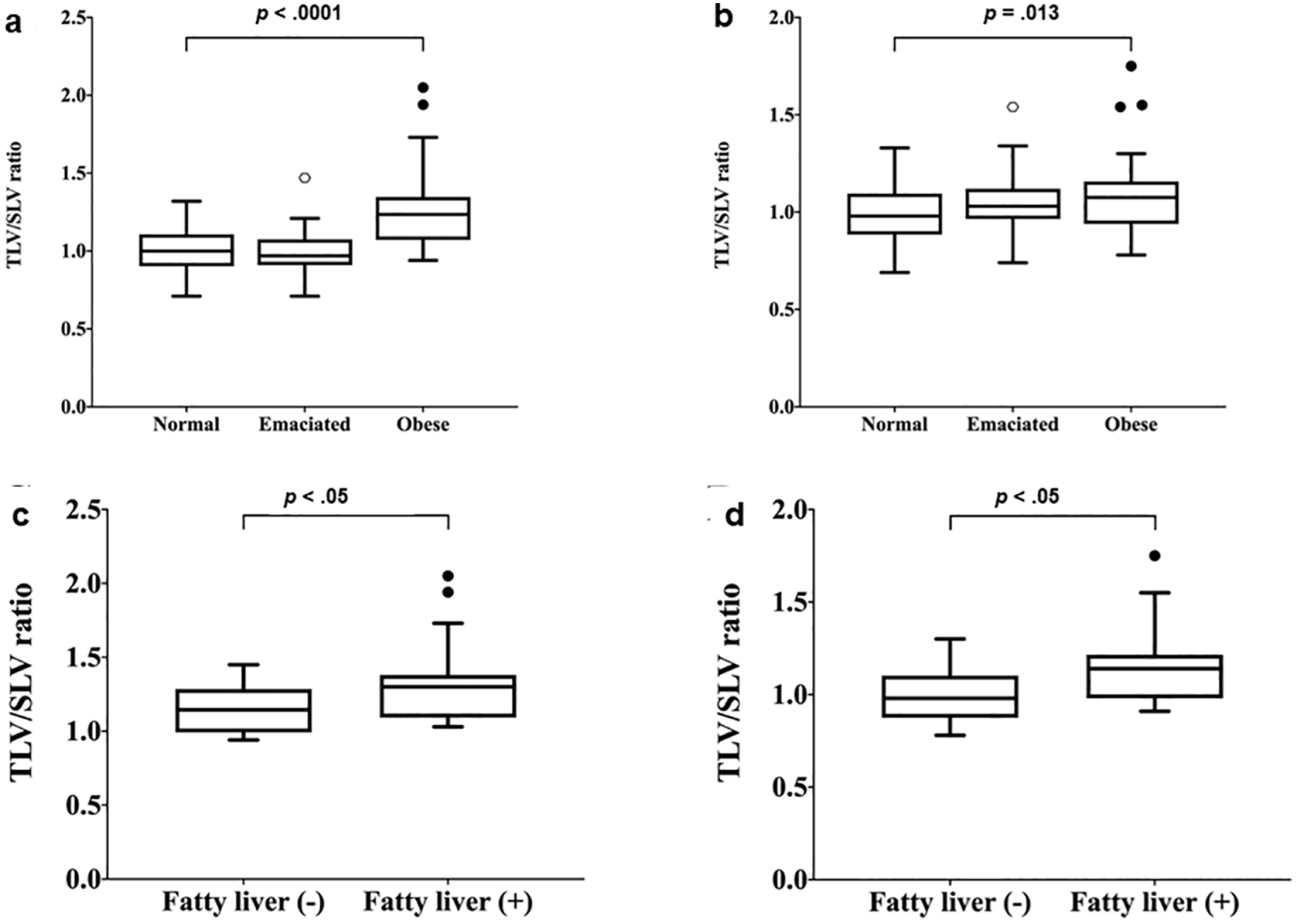
Impact of obesity and emaciation on liver volume and fatty liver status. (a, b) TLV/SLV ratios in control, emaciated, and obese subjects using Urata’s and Noda’s formulae, respectively. (c, d) TLV/SLV ratios in obese individuals with or without fatty liver. Horizontal bars represent mean ± SD. Abbreviations: TLV, total liver volume; SLV, standard liver volume. Control: subjects without obesity or emaciation (*n* = 56); Emaciation: underweight subjects (*n* = 49); Obesity: obese subjects (*n* = 28); Fatty liver: CT attenuation ≤46 HU in Couinaud segments.

The analysis included all adult (≥18 years old) subjects (*n* = 913): 836 without obesity or emaciation, 28 with obesity, and 49 with emaciation. As shown in Fig 4a and 4b, TLV/SLV ratios calculated using Urata’s and Noda’s formulae were significantly higher in obese vs. normal-weight individuals (*P* < 0.0001 and *P* = 0.013, respectively), but not significantly different in emaciated individuals (*P* = 0.11 and *P* = 0.26).

The prevalence of fatty liver was compared between 28 obese subjects and 52 age- and sex-matched non-obese/non-emaciated subjects. Mean ages were 51 vs. 52 years (*P* = 1.00), and male/female ratios were 10/18 vs. 19/33 (*P* = 1.00), respectively. Fatty liver was more common in obese subjects than in age- and sex-matched controls (57% vs. 11%, *P* < 0.01). Among obese individuals, those with fatty liver had higher TLV/SLV ratios than those without (*P* < 0.05; Fig 4c and 4d).

## Discussion

In order to resolve the long-standing inconsistencies among various SLV formula and the resulting “Tower of Babel” in the SLV literature, we designed the present study using a two-stage approach. First, we performed an external validation of 13 previously proposed SLV equations using age-range–matched cohorts, thereby placing each formula back onto its original derivation ground. Second, after identifying the most physiologically coherent equations, we examined their behavior when extrapolated into older adulthood and assessed the effects of sex and obesity/steatosis*.*

Under these unified conditions, Urata’s and Noda’s formulae consistently showed the highest accuracy. The superior performance of these equations can be explained by their adherence to allometric principles. Liver size increases subproportionally with body size, reflecting a hypoallometric scaling relationship observed both during growth and among individuals with different body weights. Such scaling behavior is more appropriately captured by BW-based power-law or BSA-proportional functions, as employed by Noda and Urata, respectively [1,3]. In contrast, SLV formulae based on linear BW relationships exhibited greater inaccuracies and lower ICCs [9,12,14].

When linear adult-derived models are extrapolated beyond their original derivation ranges, distortions in slope and intercept inevitably occur. A direct manifestation of this problem is the appearance of biologically implausible non-zero intercepts, predicting substantial liver volume at zero body mass, which reflects inappropriate extrapolation [4,6,9,11–15].

When applied beyond their original derivation ranges, Urata’s and Noda’s formulae remained accurate until the late 40s but demonstrated a systematic deviation thereafter, with a linear decline of approximately 2.7–2.8% per five years beginning at age 50. Several SLV formulae derived from cohorts spanning a wide age range up to their 90s (e.g., Vauthey, Yoshizumi, and Yu), did not explicitly consider age-related changes in liver volume. Consequently, systematic overestimation becomes apparent in older subjects because age-related hepatic involution was never incorporated into the regression framework [6–8]. More broadly, none of the previously proposed SLV equations explicitly discussed the valid application range of regression-derived models.

Importantly, this phenomenon is distinct from allometric scaling and instead reflects age-related hepatic involution, which has been inferred in prior studies based on indirect or low-resolution measurements [22,23]. These findings demonstrate that liver volume across the human lifespan reflects at least two distinct physiological processes: allometric growth during development and age-related hepatic involution during aging. Consequently, these changes cannot be adequately represented by a single regression framework in which age is treated merely as a continuous covariate without explicit consideration of age-specific physiological transitions. Indeed, although some SLV equations incorporated age as a regression variable, age was treated merely as a covariate across the entire cohort, without explicit consideration of age-related hepatic involution or definition of a valid application range [9,16].

Another noteworthy observation concerns the strength of allometric scaling across different life stages. The correlation between body size and liver volume was markedly stronger during growth than in adulthood. Urata’s original dataset, comprising individuals aged 0–27 years, yielded an exceptionally high coefficient of determination (R² = 0.96), whereas adult-focused models such as those of Vauthey (14–90 years) and Hashimoto (17–66 years) reported substantially lower R² values of 0.46 and 0.58, respectively [6,11]. This contrast indicates that the intrinsic body–liver size relationship is most tightly constrained during growth and young adulthood, whereas in later adulthood liver volume becomes increasingly influenced by additional biological variability. Such variability likely reflects non-genetic factors, including environmental and metabolic influences, and possibly epigenetic modifications.

By contrast, sex exerted only a limited influence on liver volume, whereas body composition showed more distinct effects. Sex was not a significant determinant of SLV estimation, consistent with previous autopsy and imaging studies [9,12,17,18,32]. Emaciation likewise had little influence on liver volume. In contrast, obesity was associated with increased TLV, an effect attributable primarily to the higher prevalence of hepatic steatosis, as confirmed by CT attenuation analysis. Importantly, however, an increase in liver volume does not necessarily indicate greater functional capacity, particularly when driven by fatty infiltration rather than true parenchymal hypertrophy.

In Western clinical practice, particularly in extended liver resection, the equation proposed by Vauthey et al. has been widely adopted [6]. Discrepancies among SLV equations derived from Western and Asian, particularly Japanese, populations have often been attributed to population or racial differences, with the implication that equations derived from Japanese cohorts may underestimate TLV in Western populations [6,16,18]. However, such an interpretation may be premature if differences in derivation age range, allometric model structure, and age-related hepatic involution are not adequately considered.

A large autopsy-based study demonstrated that organ-to-body weight ratios, including that of the liver, do not differ between individuals of African (n = 149) and European (n = 172) descent [32]. Although this comparison does not directly include East Asian populations, this relationship is likely to extend to European and East Asian populations, given that genetic diversity is greater within African populations than between non-African populations as a result of the out-of-Africa bottleneck [33–35].

In addition, in the present study, validation was performed in a Japanese cohort, yet the SLV equations that demonstrated no substantial bias were not confined to any single derivation population. Among the unbiased equations, several were derived from European, Japanese, and other East Asian cohorts, indicating that model validity did not segregate by population origin.

Furthermore, this observation is consistent with the meta-analysis by Johnson et al., which reported no difference in TLV normalized for body surface area between Japanese and European individuals [10].

Taken together, these findings suggest that discrepancies among SLV equations are more plausibly explained by differences in derivation age range, allometric scaling, and age-related hepatic involution than by population origin alone.

The limitations of the present study are as follows. First, although the observed age-related reduction in liver volume may have broader clinical implications, including possible contribution to the reduced tolerance of older individuals to major hepatectomy, systemic chemotherapy, or hepatotoxic drugs, it remains uncertain whether this reduction represents a physiological adaptation to aging or a manifestation of the aging process itself. Further studies integrating both volumetric and functional assessments will be necessary to clarify these relationships. Second, although age ranges were matched between our validation cohorts and the original derivation cohorts, detailed age distributions were not available for precise alignment. Finally, our cohort consisted of individuals without known liver disease, including some with obesity or emaciation, but did not include patients with pathological conditions such as ascites or sarcopenia. Therefore, our findings may not be directly applicable to individuals with extreme metabolic or fluid status alterations.

Beyond transplantation planning, accurate estimation of physiologically expected liver volume may also provide a useful baseline for individualized assessment in other areas of hepatology and liver surgery. Future studies may explore how age-aware SLV estimation could be integrated into functional or therapeutic evaluation frameworks in chronic liver disease and liver cancer.

Despite these limitations and remaining uncertainties, this study has several notable strengths. It is the first to validate multiple SLV formulae in a systematically standardized manner using age-matched external cohorts. The present study provides a reproducible platform for assessing SLV formulae on equal terms and offers guidance for appropriate extrapolation across age and body composition.

## Conclusion

Every robust scientific model ideally incorporates previous frameworks as locally valid approximations within a broader and more general theory. Just as Einstein’s general theory of relativity extended Newtonian mechanics to encompass a wider range of gravitational phenomena, the present findings refine and contextualize prior SLV formulae within a more comprehensive physiological framework. Urata’s and Noda’s equations remain valid for both sexes and reliably estimate SLV in individuals up to their late 40s. Beyond the age of 50, however, liver volume decreases in a linear manner, necessitating correction factors of approximately −2.8% (Urata) and −2.7% (Noda) for every additional five years of age. Accordingly, in individuals younger than 50 years, conventional SLV formulae may be used without modification, whereas after the age of 50, age-related decline in liver volume may be pragmatically approximated by reducing the estimated SLV according to these correction rates (e.g., adjusted SLV ≈ conventional SLV × [1 − (age − 50) × 0.028/5] for Urata’s formula). Such an approach may improve the accuracy of graft planning while avoiding unnecessary proliferation of new formulae and instead reinterpreting existing models within an age-aware physiological framework.

## Supporting information

S1 File Original_data_anonymous. (XLSX)
