# Supplementary material for: Age-range–matched external validation of standard liver volume equations: Methodological re-evaluation of 13 regression models
Source: PLoS One. 2026 Jul 8;21(7):e0351983. doi: 10.1371/journal.pone.0351983 (PMC13345290; doi:10.1371/journal.pone.0351983)
Supplement: Attachment — Submitted filename: Response yp Reviewers.docx [file pone.0351983.s003.docx]

We thank the Academic Editor and the editorial office for their careful evaluation of our manuscript and for the opportunity to revise it. We have revised the manuscript and supporting files in accordance with the journal requirements and reviewer comments. Our point-by-point responses are provided below.

**Responses to Journal (Editorial) Requirements**

**1. PLOS ONE style requirements and file naming**

**Response:**

We have revised the manuscript format and file naming according to the PLOS ONE style requirements.

**2. Code sharing**

**Response:**

No custom software was developed for this study. Statistical analyses were performed using SAS, and the statistical procedures are described in the Methods section to ensure reproducibility.

**3. Funding statement**

**Response:**

We have removed funding-related text from the manuscript outside the Funding section. The Funding Statement remains unchanged as follows:
“The authors received no specific funding for this work.”

**4. Figure captions**

**Response:**

We have revised the figure captions so that each figure has a separate, self-contained caption in accordance with PLOS ONE formatting requirements. Specifically, we standardized figure citations using the journal-preferred “Fig 1” format and ensured that each caption includes complete descriptions and abbreviations.

**5. Language of Supporting Information**

**Response:**

The dataset provided as Supporting Information is already fully in English. The non-English content identified corresponds to the original ethics approval document issued in Japanese. As required during the initial submission, we have provided an official English-language certificate of approval issued by our institution as Supporting Information. This document represents the formal and certified version for international use.

Therefore, we have retained the official English version and removed the Japanese version to comply with the journal’s language requirements.

**6. Data anonymization and availability**

**Response:**

We have revised the Supporting Information dataset to ensure full anonymization in accordance with PLOS data sharing policies. Specifically, exact age values have been replaced with categorized age groups to eliminate any potential risk of participant identification.

All other potentially identifying variables had already been removed in the original submission. The revised dataset therefore contains no direct or indirect identifiers and fully complies with participant privacy protection requirements.

In addition, we have confirmed with our institutional ethics committee that the anonymized dataset can be shared publicly in accordance with applicable regulations.

**Response to the comments from the reviewer 1**

**Comment 1. *The definition of “healthy individuals” should be clarified more clearly.***
*The manuscript repeatedly describes the validation cohort as “healthy individuals.” However, the actual cohort includes living donor candidates, patients undergoing elective laparoscopic cholecystectomy, and patients who underwent contrast-enhanced CT for acute abdomen, trauma, urologic disease, gonadal disease, and other conditions. In the current form, it is not clear how “healthy” was defined in this study and what exclusion criteria were applied. Please explain in more detail how subjects with possible liver dysfunction, chronic liver disease, nutritional problems, or other conditions that may affect liver volume were assessed and excluded. This point is important because the reliability of the reference TLV depends on the appropriateness of the study population.*

**Response:**

We thank the reviewer for this important and insightful comment.

We agree that the term “healthy individuals” may not have been sufficiently defined in the original manuscript and could lead to ambiguity. We have therefore revised the manuscript to clarify the definition of the validation cohort and the inclusion/exclusion criteria in greater detail.

Specifically, the cohort consisted of three groups:

1 Living donor candidates, who underwent extensive clinical, biochemical, and imaging evaluation and had no evidence of liver disease, including steatosis. These individuals can be considered physiologically normal by definition.
2 Adult patients undergoing elective laparoscopic cholecystectomy for benign gallbladder disease. These patients were included consecutively to minimize selection bias. Although minor laboratory abnormalities may have been present in some cases, individuals with known chronic liver disease, malignancy, or conditions affecting liver morphology were excluded. We intentionally adopted this consecutive inclusion strategy to enhance generalizability, accepting a trade-off between strict physiological normality and real-world applicability.
3 Pediatric patients who underwent contrast-enhanced CT for conditions unrelated to hepatobiliary or malignant disease. Cases with any known liver pathology were excluded.

Accordingly, we have revised the wording in the manuscript to avoid the potentially misleading term “healthy individuals” and instead describe the cohort as a “clinically normal population without known liver disease.”

We believe this approach provides a more realistic and generalizable reference for CT-measured TLV, while maintaining sufficient biological validity for the purpose of external validation.

The revised parts are as follows:

Page 2, lines 13-14 (new), page 6, lines 17-18 (new), and page 20, line 6 (new)

individuals without known liver disease

Page 7, line 22-page 8, line 5 (new)

Individuals with known chronic liver disease, malignancy, or structural liver abnormalities were excluded. Living donor candidates were considered physiologically normal based on comprehensive clinical evaluation, including laboratory testing and imaging. For non-donor subjects, we adopted a consecutive inclusion strategy to minimize selection bias, accepting a trade-off between strict physiological normality and generalizability.

**Comment 2.** ***More methodological detail is needed for CT volumetry.*** *The manuscript states that TLV was measured using contrast-enhanced CT and three-dimensional image analysis software, but the actual measurement procedure is not sufficiently described. Please clarify whether liver segmentation was manual, semi-automatic, or automatic, whether major intrahepatic vessels were included or excluded, and how focal hepatic lesions or other factors potentially affecting liver volume were handled. Since this study is essentially a validation study, the reliability of the CT-based reference standard should be described more clearly.*

**Response:**

We have revised the Methods section to provide additional detail on the measurement procedure. TLV was measured using contrast-enhanced CT images with 1-mm slice thickness and three-dimensional image analysis software (Synapse VINCENT; Fujifilm Corporation, Tokyo, Japan). Liver segmentation was performed using a semi-automated approach, in which hepatic contours and intrahepatic vascular structures were initially detected automatically and subsequently reviewed and, if necessary, manually adjusted by experienced operators.

Major intrahepatic vessels were excluded from the volume calculation, and focal hepatic lesions, all of which were benign in this cohort, were also excluded. All segmentations were visually confirmed to ensure accuracy.

Although the software used in this study allows detailed segmental volumetry, the present analysis focused exclusively on total liver volume (TLV), and segmental volume measurements were not used. This point has been clarified in the manuscript to avoid potential confusion.

We believe that these additions clarify the reliability of CT-based volumetry as the reference standard in the present validation study.

The revision is as follows:

Page 8, lines 8-18 (new)

TLV was measured using contrast-enhanced CT images with 1-mm slice thickness and three-dimensional image analysis software (Synapse VINCENT; Fujifilm Corporation, Tokyo, Japan). Liver segmentation was performed using a semi-automated approach, in which hepatic contours and intrahepatic vascular structures were initially detected automatically and subsequently reviewed and, if necessary, manually adjusted by experienced operators. Major intrahepatic vessels were excluded from volume calculation, and focal hepatic lesions, all of which were benign in this cohort, were also excluded. Although the software allows detailed segmental volumetry, the present study focused exclusively on TLV, and segmental volume measurements were not used in the analysis.

**Comment 3: *The discussion on race or population differences should be shortened and toned down.***

*The discussion about racial explanation is interesting, but it is somewhat too long and too assertive compared with the main message of this study. Since the present analysis was performed in a single Japanese cohort, the manuscript should be more careful when making broader statements about the absence of population-related differences. A shorter and more focused discussion would improve the balance of the paper.*

**Response:**

We thank the reviewer for this important and thoughtful comment.

We agree that the original discussion on population and racial differences was overly long and somewhat assertive relative to the main focus of the present study. Accordingly, we have substantially shortened and revised this section to improve balance and clarity.

In the revised manuscript, we have removed broader statements regarding human genetic diversity and focused the discussion on evidence directly relevant to liver volume. Specifically, we retained (i) the autopsy-based study demonstrating no difference in liver-to-body weight ratios between African and European individuals, (ii) our own validation results showing that unbiased equations were not confined to a single derivation population, and (iii) the meta-analysis by Johnson et al. reporting no difference in TLV normalized for body surface area between Japanese and European individuals.

At the same time, we have moderated the tone of the discussion and avoided overly definitive statements. The revised text now emphasizes that discrepancies among SLV equations are more plausibly explained by differences in derivation age range, allometric scaling, and age-related hepatic involution, rather than attributing them primarily to population origin.

We believe that these revisions address the reviewer’s concern while preserving the methodological implications of our findings.

The revised corresponding part of Discussion section is as follows:

Page 18, line 4-page 19, line 13 (new)

In Western clinical practice, particularly in extended liver resection, the equation proposed by Vauthey et al. has been widely adopted [6]. Discrepancies among SLV equations derived from Western and Asian, particularly Japanese, populations have often been attributed to population or racial differences, with the implication that equations derived from Japanese cohorts may underestimate TLV in Western populations [6,16,18]. However, such an interpretation may be premature if differences in derivation age range, allometric model structure, and age-related hepatic involution are not adequately considered.

A large autopsy-based study demonstrated that organ-to-body weight ratios, including that of the liver, do not differ between individuals of African (n = 149) and European (n = 172) descent [32]. Although this comparison does not directly include East Asian populations, this relationship is likely to extend to European and East Asian populations, given that genetic diversity is greater within African populations than between non-African populations as a result of the out-of-Africa bottleneck [33].

In addition, in the present study, validation was performed in a Japanese cohort, yet the SLV equations that demonstrated no substantial bias were not confined to any single derivation population. Among the unbiased equations, several were derived from European, Japanese, and other East Asian cohorts, indicating that model validity did not segregate by population origin.

Furthermore, this observation is consistent with the meta-analysis by Johnson et al., which reported no difference in TLV normalized for body surface area between Japanese and European individuals [10].

Taken together, these findings suggest that discrepancies among SLV equations are more plausibly explained by differences in derivation age range, allometric scaling, and age-related hepatic involution than by population origin alone.

**Comment 4.** ***There is an internal contradiction in the Data Availability Statement.***

*In the manuscript, the Data Availability Statement says that all relevant data are within the paper and its Supporting Information files. However, in the later Data availability section, the authors state that the raw data will be made available upon due request from the corresponding author. These two statements are not consistent and should be corrected*.

**Response:**

We agree that the original manuscript contained contradictory statements regarding data availability. To resolve this issue, we removed the statement indicating that raw data would be made available upon request from the corresponding author.

The revised manuscript now consistently states that all relevant data are available within the paper and its Supporting Information files, in accordance with PLOS ONE data sharing policies.

**Minor comment:** *Some expressions are too rhetorical for a scientific paper. For example, the “Tower of Babel” expression in the Introduction, the Einstein/Newton analogy in the Conclusion, and the quotation from T. S. Eliot may be too literary for this type of article. A more neutral style would be preferable.*

**Response:**

We thank the reviewer for this thoughtful suggestion.

We agree that some expressions in the original manuscript were more conceptual and metaphorical than is typical for a scientific article. However, our intention was to communicate complex methodological relationships in a manner that would be intuitively understandable to readers, particularly the idea that previously proposed SLV equations should not necessarily be regarded as mutually exclusive or competing constructs.

Accordingly, we revised and moderated several expressions to improve the scientific tone of the manuscript. Specifically, the quotation from T. S. Eliot was removed, and the “Tower of Babel” expression was slightly softened. However, the analogy between classical and more general physical theories was retained in a moderated form because it conveys the central conceptual message of the study, namely that broader physiological frameworks may incorporate previously proposed equations as locally valid approximations rather than replacing them entirely.

We hope that these revisions improve the overall balance and readability of the manuscript while preserving its conceptual clarity. We would, of course, be happy to make further modifications should the editor consider additional changes necessary.

The revisions are as follows:

Page 5, line 1 (new).

a situation that has created what may be described as a “Tower of Babel” in the SLV literature.

Page 21, line 6 (new).

Every robust scientific model ideally incorporates previous frameworks as locally valid approximations within a broader and more general theory.

Page 20, lines 11-14 (old) deleted

In essence, this study returns to the foundations of SLV estimation with a broader and more integrative perspective. As T. S. Eliot wrote, *“the end of all our exploring will be to arrive where we started and know the place for the first time.”*

Page 22, lines 2-3 (new).

and instead reinterpreting existing models within an age-aware physiological framework.

**Response to the comments from the reviewer 2**

**Comment 1.** *It is mentioned in the conclusion (lines 9-10, page 10) that "incorporation of age adjustment into clinical practice may improve transplantation planning." You can add 1-2 sentences here to specify how to do this. For example, would it be recommended to develop a new formula that includes an age factor, or would it be recommended to multiply directly by an age-corrected factor (e.g., every 5 years after >50 years) after using the Urata/Noda formula? Providing a concrete calculation example will make the recommendations more operational.*

**Response:**

We thank the reviewer for this constructive suggestion.

We agree that a more operational description would improve the clinical interpretability of our findings. However, the intention of the present study was not to propose yet another SLV equation, but rather to reinterpret existing formulae within an age-aware physiological framework.

According to the reviewer’s suggestion, we added a brief practical example in the Conclusion describing how age-related correction may be pragmatically applied after the age of 50 while avoiding unnecessary proliferation of new formulae.

Page 21, line 14-page 22, line 2 (new).

Beyond the age of 50, however, liver volume decreases in a linear manner, necessitating correction factors of approximately −2.8% (Urata) and −2.7% (Noda) for every additional five years of age. Accordingly, in individuals younger than 50 years, conventional SLV formulae may be used without modification, whereas after the age of 50, age-related decline in liver volume may be pragmatically approximated by reducing the estimated SLV according to these correction rates (e.g., adjusted SLV ≈ conventional SLV × [1 − (age − 50) × 0.028/5] for Urata’s formula). Such an approach may improve the accuracy of graft planning while avoiding unnecessary proliferation of new formulae.

Instead, the following description was deleted.

Page 20 lines 8-10 (old).

Incorporating these age-related modifications into clinical practice may improve the accuracy of graft planning and enhance outcomes in liver transplantation.

**Comment 2.** *In the discussion of the linear decrease in liver volume with age (lines 1-13 on page 14), the potential clinical implications may be briefly discussed." Might this physiological reduction in "liver reserve", for example, partly explain the phenomenon of reduced tolerance of elderly patients to hepatectomy, chemotherapy, or certain drugs such as acetaminophen? This will link the findings to broader issues in liver surgery and hepatology.*

**Response:**

We thank the reviewer for this insightful suggestion.

We agree that the observed age-related decline in liver volume may have broader clinical implications beyond SLV estimation itself. Accordingly, we have expanded the limitation section to discuss the possibility that reduced liver volume in older individuals may partly contribute to decreased tolerance to major hepatectomy, systemic chemotherapy, or hepatotoxic drugs.

At the same time, we emphasized that it remains uncertain whether the observed decline represents a physiological adaptation to aging or a manifestation of the aging process itself, and that further studies integrating both volumetric and functional assessments will be necessary to clarify these relationships.

Accordingly, the limitation part is modified in the revised manuscript as follows:

Page 19, line 14-page 20, line 3 (new)

The limitations of the present study are as follows. First, although the observed age-related reduction in liver volume may have broader clinical implications, including possible contribution to the reduced tolerance of older individuals to major hepatectomy, systemic chemotherapy, or hepatotoxic drugs, it remains uncertain whether this reduction represents a physiological adaptation to aging or a manifestation of the aging process itself. Further studies integrating both volumetric and functional assessments will be necessary to clarify these relationships.

**Comment 3.** *In discussing study limitations or future directions (lines 1-10 on page 18), add a paragraph stating that the precise SLV model developed in this study provides a "baseline" or "frame of reference" for individualized treatment evaluation in other liver diseases. "The age-adjusted SLV established in this study provides a more precise baseline for individualized assessment in liver cancer therapy. For example, an accurate expected total liver volume is critical when assessing the impact of targeted or immunotherapy on liver tumor burden. Future studies could explore the integration of such accurate SLV models into the evaluation system of treatment response in HCC, as highlighted in the discussion on overcoming the bottleneck in HCC clinical trials, accurate baseline assessment is a key part of optimizing treatment strategy [DOI: 10.1007/s12072-025-10799-2].*

**Response:**

We thank the reviewer for this insightful suggestion.

We agree that accurate age-aware SLV estimation may have implications beyond transplantation planning alone. However, because the present study focused primarily on methodological validation of SLV equations rather than therapeutic assessment in HCC, we chose to address this point in a concise and exploratory manner.

Accordingly, we added a brief paragraph suggesting that physiologically adjusted SLV estimation may provide a useful baseline or frame of reference for individualized assessment in other areas of hepatology and liver surgery, including chronic liver disease and liver cancer.

Revisions are as follows:

Page 20, lines 10-14 (new)

Beyond transplantation planning, accurate estimation of physiologically expected liver volume may also provide a useful baseline for individualized assessment in other areas of hepatology and liver surgery. Future studies may explore how age-aware SLV estimation could be integrated into functional or therapeutic evaluation frameworks in chronic liver disease and liver cancer.

**Comment 4.** *"In addition, accurate SLV is critical for the field of liver transplantation, not only for graft size matching, but also for assessing the 'reserve pool' of liver function recovery after transplantation. In Settings involving ischemia-reperfusion injury (e.g., early after transplantation), the ratio of actual to standard liver volume may serve as an emerging parameter for assessing the balance between liver injury and regenerative capacity. It is worth exploring the potential value of SLV in predicting or assessing the outcome of liver stress conditions such as ischemia-reperfusion injury in the future [PMID: 41582651]."*

**Response:**

We agree that accurate SLV estimation may potentially contribute to broader discussions regarding hepatic reserve and regenerative capacity in various clinical settings. However, because the present study focused primarily on methodological validation of SLV equations and age-related volumetric changes, we considered a detailed discussion of ischemia-reperfusion injury to be beyond the scope of the current manuscript.

Nevertheless, we agree that the relationship between morphologically estimated liver reserve and functional recovery after hepatic injury or transplantation may represent an interesting direction for future investigation. In response to the reviewer’s previous comment, we added a brief paragraph discussing the broader relevance of physiologically adjusted SLV estimation in hepatology and liver surgery, which we believe this also addresses important perspective.

**Comment 5*.*** *Figure 1: Since there are 13 subgraphs, although the current layout is clear, when the key findings are mentioned in the text (such as the description on page 9 of the existence of "two independent clusters" of the formula of Yoshizumi, Johnson, and Yu), consider slightly highlighting the two clusters in the corresponding subgraphs (Figure 1d-f) with dashed boxes or different colors. To help readers understand this important finding more intuitively.*

**Response:**

We agree that the clustering pattern observed in Yoshizumi’s, Johnson’s, and Yu’s formulae represents an important methodological finding. However, because Bland–Altman plots are highly dependent on visual interpretation, we preferred to avoid additional graphical highlighting (e.g., dashed boxes or color overlays), which might unintentionally overemphasize or visually bias interpretation of the scatter pattern.

Instead, we clarified this point in the figure legend by explicitly stating that visually distinct clustering contributed to attenuation of the overall proportional bias estimates. The revisions are as follows:

Page 35, lines 13–16 (new)

The apparent low proportional bias observed in some equations, particularly Yoshizumi’s, Johnson’s, and Yu’s formulae, should be interpreted cautiously, as visually distinct clustering patterns contributed to attenuation of the overall proportional bias estimates.

**Comment 6.** *Terminology unification: TLV (total liver volume) is mainly used as the gold standard in this paper. When referring to "CT-measured TLV" in the abstract, results, etc., it can be simplified to "measured TLV" or "CT-volumetry" to keep it concise.*

**Response:**

We thank the reviewer for this helpful suggestion.

We agree that excessive repetition of “CT-measured TLV” may reduce readability. Accordingly, we simplified selected instances after the initial definition where the reference standard was already clear from the context.

However, because the distinction between estimated SLV and CT-based reference volumetry is central to the present study, we retained the term “CT-measured TLV” in contexts where clarification of the reference standard was considered important.

The revised part is as follows:

Page 11, line 17 (new).

Overall, Urata’s, Noda’s, and Yang’s formulae, which predict measured TLV by BSA or BW, were identified as potential candidates for the further examination;
