# Supplementary material for: Age-range–matched external validation of standard liver volume equations: Methodological re-evaluation of 13 regression models
Source: PLoS One. 2026 Jul 8;21(7):e0351983. doi: 10.1371/journal.pone.0351983 (PMC13345290; doi:10.1371/journal.pone.0351983)
Supplement: Peer review history

# Peer review: Decision Letter 0

**DOI:** 10.1371/journal.pone.0351983.r001

**Authors:** Yun-Wen Zheng

29 Apr 2026

PONE-D-26-09031

Age-range–matched external validation of standard liver volume equations: methodological re-evaluation of 13 regression models

PLOS One

Dear Dr. Imamura,

Thank you for submitting your manuscript to PLOS ONE. After careful consideration, we feel that it has merit but does not fully meet PLOS ONE’s publication criteria as it currently stands. Therefore, we invite you to submit a revised version of the manuscript that addresses the points raised during the review process.

This manuscript carries out an external validation of thirteen standard liver volume (SLV) formulae through age-range matching. This clarifies key controversies in SLV estimation and offers methodological and clinical implications for liver surgery and transplantation. While the study has clear scientific merit and innovative value, methodological clarity, content refinement, consistency, writing style and clinical elaboration require revision, as the reviewers pointed out. authors must follow all required changes and are strongly encouraged to implement all recommended revisions to improve clarity, rigor, and clinical impact.

Please submit your revised manuscript by Jun 13 2026 11:59PM. If you will need more time than this to complete your revisions, please reply to this message or contact the journal office at plosone@plos.org. When you're ready to submit your revision, log on to https://www.editorialmanager.com/pone/ and select the 'Submissions Needing Revision' folder to locate your manuscript file.

Please include the following items when submitting your revised manuscript:

- A letter that responds to each point raised by the academic editor and reviewer(s). You should upload this letter as a separate file labeled 'Response to Reviewers'.
- A marked-up copy of your manuscript that highlights changes made to the original version. You should upload this as a separate file labeled 'Revised Manuscript with Track Changes'.
- An unmarked version of your revised paper without tracked changes. You should upload this as a separate file labeled 'Manuscript'.

If you would like to make changes to your financial disclosure, please include your updated statement in your cover letter. Guidelines for resubmitting your figure files are available below the reviewer comments at the end of this letter.

If applicable, we recommend that you deposit your laboratory protocols in protocols.io to enhance the reproducibility of your results. Protocols.io assigns your protocol its own identifier (DOI) so that it can be cited independently in the future. For instructions see: https://journals.plos.org/plosone/s/submission-guidelines#loc-laboratory-protocols. Additionally, PLOS ONE offers an option for publishing peer-reviewed Lab Protocol articles, which describe protocols hosted on protocols.io. Read more information on sharing protocols at https://plos.org/protocols?utm_medium=editorial-email&utm_source=authorletters&utm_campaign=protocols.

As the corresponding author, your ORCID iD is verified in the submission system and will appear in the published article. PLOS supports the use of ORCID, and we encourage all coauthors to register for an ORCID iD and use it as well. Please encourage your coauthors to verify their ORCID iD within the submission system before final acceptance, as unverified ORCID iDs will not appear in the published article. *Only*  the individual author can complete the verification step; PLOS staff *cannot*  verify ORCID iDs on behalf of authors.

We look forward to receiving your revised manuscript.

Kind regards,

Yun-Wen Zheng

Academic Editor

PLOS One

Journal Requirements:

When submitting your revision, we need you to address these additional requirements.

1. Please ensure that your manuscript meets PLOS ONE's style requirements, including those for file naming. The PLOS ONE style templates can be found at

https://journals.plos.org/plosone/s/file?id=wjVg/PLOSOne_formatting_sample_main_body.pdf and

https://journals.plos.org/plosone/s/file?id=ba62/PLOSOne_formatting_sample_title_authors_affiliations.pdf

2. Please note that PLOS One has specific guidelines on code sharing for submissions in which author-generated code underpins the findings in the manuscript. In these cases, all author-generated code must be made available without restrictions upon publication of the work. Please review our guidelines at https://journals.plos.org/plosone/s/materials-and-software-sharing#loc-sharing-code and ensure that your code is shared in a way that follows best practice and facilitates reproducibility and reuse.

3. Thank you for stating the following in the Acknowledgments Section of your manuscript:

“The author(s) received no specific funding for this work.”

We note that you have provided funding information that is not currently declared in your Funding Statement. However, funding information should not appear in the Acknowledgments section or other areas of your manuscript. We will only publish funding information present in the Funding Statement section of the online submission form.

Please remove any funding-related text from the manuscript and let us know how you would like to update your Funding Statement. Currently, your Funding Statement reads as follows:

“The author(s) received no specific funding for this work.”

Please include your amended statements within your cover letter; we will change the online submission form on your behalf.

4. Please include a separate caption for each figure in your manuscript.

5. Thank you for providing your underlying data as Supporting Information.

We note that the data set contains text or data that is not in English. Please note that PLOS is an English-language publisher, so we require data sets to be provided in English as well. Please upload an English-language version of your data set.

This will also allow us to determine if your data follows PLOS standards per our Data Availability policy here: https://journals.plos.org/plosone/s/data-availability

6. We note that there is identifying data in the Supporting Information file “Original_data.xlsx”. Due to the inclusion of these potentially identifying data, we have removed this file from your file inventory. Prior to sharing human research participant data, authors should consult with an ethics committee to ensure data are shared in accordance with participant consent and all applicable local laws.

Data sharing should never compromise participant privacy. It is therefore not appropriate to publicly share personally identifiable data on human research participants. The following are examples of data that should not be shared:

-Name, initials, physical address

-Ages more specific than whole numbers

-Internet protocol (IP) address

-Specific dates (birth dates, death dates, examination dates, etc.)

-Contact information such as phone number or email address

-Location data

-ID numbers that seem specific (long numbers, include initials, titled “Hospital ID”) rather than random (small numbers in numerical order)

Data that are not directly identifying may also be inappropriate to share, as in combination they can become identifying. For example, data collected from a small group of participants, vulnerable populations, or private groups should not be shared if they involve indirect identifiers (such as sex, ethnicity, location, etc.) that may risk the identification of study participants.

Additional guidance on preparing raw data for publication can be found in our Data Policy (https://journals.plos.org/plosone/s/data-availability#loc-human-research-participant-data-and-other-sensitive-data) and in the following article: http://www.bmj.com/content/340/bmj.c181.long.

Please remove or anonymize all personal information “Age”, ensure that the data shared are in accordance with participant consent, and re-upload a fully anonymized data set. Please note that spreadsheet columns with personal information must be removed and not hidden as all hidden columns will appear in the published file.

7. If the reviewer comments include a recommendation to cite specific previously published works, please review and evaluate these publications to determine whether they are relevant and should be cited. There is no requirement to cite these works unless the editor has indicated otherwise.

Additional Editor Comments:

This manuscript carries out an external validation of thirteen standard liver volume (SLV) formulae through age-range matching. This clarifies key controversies in SLV estimation and offers methodological and clinical implications for liver surgery and transplantation. While the study has clear scientific merit and innovative value, methodological clarity, content refinement, consistency, writing style and clinical elaboration require revision, as the reviewers pointed out.

[Note: HTML markup is below. Please do not edit.]

Reviewers' comments:

Reviewer's Responses to Questions

**Comments to the Author**

1. Is the manuscript technically sound, and do the data support the conclusions?

The manuscript must describe a technically sound piece of scientific research with data that supports the conclusions. Experiments must have been conducted rigorously, with appropriate controls, replication, and sample sizes. The conclusions must be drawn appropriately based on the data presented.

Reviewer #1: Partly

Reviewer #2: Yes

**********

2. Has the statistical analysis been performed appropriately and rigorously?

Reviewer #1: Yes

Reviewer #2: Yes

**********

3. Have the authors made all data underlying the findings in their manuscript fully available?

The PLOS Data policy requires authors to make all data underlying the findings described in their manuscript fully available without restriction, with rare exception (please refer to the Data Availability Statement in the manuscript PDF file). The data should be provided as part of the manuscript or its supporting information, or deposited to a public repository. For example, in addition to summary statistics, the data points behind means, medians and variance measures should be available. If there are restrictions on publicly sharing data—e.g. participant privacy or use of data from a third party—those must be specified.

Reviewer #1: No

Reviewer #2: Yes

**********

4. Is the manuscript presented in an intelligible fashion and written in standard English?

PLOS ONE does not copyedit accepted manuscripts, so the language in submitted articles must be clear, correct, and unambiguous. Any typographical or grammatical errors should be corrected at revision, so please note any specific errors here.

Reviewer #1: No

Reviewer #2: Yes

**********

5. Review Comments to the Author

Please use the space provided to explain your answers to the questions above. You may also include additional comments for the author, including concerns about dual publication, research ethics, or publication ethics. (Please upload your review as an attachment if it exceeds 20,000 characters)

Reviewer #1: The authors examined the agreement between estimated standard liver volume (SLV) and CT-measured total liver volume (TLV) across 13 previously published formulae, using an age-range-matched external validation approach. The main idea of evaluating each formula only within its original derivation age range is important, and the study has merit. However, several points should be clarified before the manuscript can be considered further.

Major comments

1. The definition of “healthy individuals” should be clarified more clearly.

The manuscript repeatedly describes the validation cohort as “healthy individuals.” However, the actual cohort includes living donor candidates, patients undergoing elective laparoscopic cholecystectomy, and patients who underwent contrast-enhanced CT for acute abdomen, trauma, urologic disease, gonadal disease, and other conditions. In the current form, it is not clear how “healthy” was defined in this study and what exclusion criteria were applied. Please explain in more detail how subjects with possible liver dysfunction, chronic liver disease, nutritional problems, or other conditions that may affect liver volume were assessed and excluded. This point is important because the reliability of the reference TLV depends on the appropriateness of the study population.

2. More methodological detail is needed for CT volumetry.

The manuscript states that TLV was measured using contrast-enhanced CT and three-dimensional image analysis software, but the actual measurement procedure is not sufficiently described. Please clarify whether liver segmentation was manual, semi-automatic, or automatic, whether major intrahepatic vessels were included or excluded, and how focal hepatic lesions or other factors potentially affecting liver volume were handled. Since this study is essentially a validation study, the reliability of the CT-based reference standard should be described more clearly.

3. The discussion on race or population differences should be shortened and toned down.

The discussion about racial explanation is interesting, but it is somewhat too long and too assertive compared with the main message of this study. Since the present analysis was performed in a single Japanese cohort, the manuscript should be more careful when making broader statements about the absence of population-related differences. A shorter and more focused discussion would improve the balance of the paper.

4. There is an internal contradiction in the Data Availability Statement.

In the manuscript, the Data Availability Statement says that all relevant data are within the paper and its Supporting Information files. However, in the later Data availability section, the authors state that the raw data will be made available upon due request from the corresponding author. These two statements are not consistent and should be corrected.

Minor comment

Some expressions are too rhetorical for a scientific paper. For example, the “Tower of Babel” expression in the Introduction, the Einstein/Newton analogy in the Conclusion, and the quotation from T. S. Eliot may be too literary for this type of article. A more neutral style would be preferable.

Reviewer #2: This study innovatively employed an external validation strategy of "age range matching" to systematically evaluate 13 standard liver volume (SLV) formulas. The "Tower of Babel" contradictory conclusions in the previous literature were successfully attributed to the fundamental methodological defect of ignoring the age range of the original derivation of the formula. The Urata (proportional function of body surface area) and Noda (power function of body weight) formulas were found to be most accurate in their original age range and quantified for the first time the linear decline in liver volume after age 50 ata rate of 2.7-2.8% per 5 years. This work not only clarifies the long-standing controversy about SLV estimation, but also establishes a generalizable paradigm for the external validation of regression models, which has direct guiding value for the accurate evaluation of liver transplantation and liver surgery. However, there are still some parts of the article that need to be revised before publication, as follows:

1. It is mentioned in the conclusion (lines 9-10, page 10) that "incorporation of age adjustment into clinical practice may improve transplantation planning." You can add 1-2 sentences here to specify how to do this. For example, would it be recommended to develop a new formula that includes an age factor, or would it be recommended to multiply directly by an age-corrected factor (e.g., every 5 years after >50 years) after using the Urata/Noda formula? Providing a concrete calculation example will make the recommendations more operational.

"2. In the discussion of the linear decrease in liver volume with age (lines 1-13 on page 14), the potential clinical implications may be briefly discussed." Might this physiological reduction in "liver reserve", for example, partly explain the phenomenon of reduced tolerance of elderly patients to hepatectomy, chemotherapy, or certain drugs such as acetaminophen? This will link the findings to broader issues in liver surgery and hepatology.

3. In discussing study limitations or future directions (lines 1-10 on page 18), add a paragraph stating that the precise SLV model developed in this study provides a "baseline" or "frame of reference" for individualized treatment evaluation in other liver diseases. "The age-adjusted SLV established in this study provides a more precise baseline for individualized assessment in liver cancer therapy. For example, an accurate expected total liver volume is critical when assessing the impact of targeted or immunotherapy on liver tumor burden. Future studies could explore the integration of such accurate SLV models into the evaluation system of treatment response in HCC, as highlighted in the discussion on overcoming the bottleneck in HCC clinical trials, accurate baseline assessment is a key part of optimizing treatment strategy [DOI: 10.1007/s12072-025-10799-2].

4. "In addition, accurate SLV is critical for the field of liver transplantation, not only for graft size matching, but also for assessing the 'reserve pool' of liver function recovery after transplantation. In Settings involving ischemia-reperfusion injury (e.g., early after transplantation), the ratio of actual to standard liver volume may serve as an emerging parameter for assessing the balance between liver injury and regenerative capacity. It is worth exploring the potential value of SLV in predicting or assessing the outcome of liver stress conditions such as ischemia-reperfusion injury in the future [PMID: 41582651]."

5. Figure 1: Since there are 13 subgraphs, although the current layout is clear, when the key findings are mentioned in the text (such as the description on page 9 of the existence of "two independent clusters" of the formula of Yoshizumi, Johnson, and Yu), consider slightly highlighting the two clusters in the corresponding subgraphs (Figure 1d-f) with dashed boxes or different colors. To help readers understand this important finding more intuitively.

6. Terminology unification: TLV (total liver volume) is mainly used as the gold standard in this paper. When referring to "CT-measured TLV" in the abstract, results, etc., it can be simplified to "measured TLV" or "CT-volumetry" to keep it concise.

**********

6. PLOS authors have the option to publish the peer review history of their article (what does this mean?). If published, this will include your full peer review and any attached files.

If you choose “no”, your identity will remain anonymous but your review may still be made public.

**Do you want your identity to be public for this peer review?**  For information about this choice, including consent withdrawal, please see our Privacy Policy.

Reviewer #1: No

Reviewer #2: No

**********

[NOTE: If reviewer comments were submitted as an attachment file, they will be attached to this email and accessible via the submission site. Please log into your account, locate the manuscript record, and check for the action link "View Attachments". If this link does not appear, there are no attachment files.]

To ensure your figures meet our technical requirements, please review our figure guidelines: https://journals.plos.org/plosone/s/figures

You may also use PLOS’s free figure tool, NAAS, to help you prepare publication quality figures: https://journals.plos.org/plosone/s/figures#loc-tools-for-figure-preparation.

NAAS will assess whether your figures meet our technical requirements by comparing each figure against our figure specifications.

# Author comment: Author response to Decision Letter 1

**DOI:** 10.1371/journal.pone.0351983.r002

17 May 2026

We thank the Academic Editor and the editorial office for their careful evaluation of our manuscript and for the opportunity to revise it. We have revised the manuscript and supporting files in accordance with the journal requirements and reviewer comments. Our point-by-point responses are provided below.

Responses to Journal (Editorial) Requirements

1. PLOS ONE style requirements and file naming

Response:

We have revised the manuscript format and file naming according to the PLOS ONE style requirements.

2. Code sharing

Response:

No custom software was developed for this study. Statistical analyses were performed using SAS, and the statistical procedures are described in the Methods section to ensure reproducibility.

3. Funding statement

Response:

We have removed funding-related text from the manuscript outside the Funding section. The Funding Statement remains unchanged as follows:

“The authors received no specific funding for this work.”

4. Figure captions

Response:

We have revised the figure captions so that each figure has a separate, self-contained caption in accordance with PLOS ONE formatting requirements. Specifically, we standardized figure citations using the journal-preferred “Fig 1” format and ensured that each caption includes complete descriptions and abbreviations.

5. Language of Supporting Information

Response:

The dataset provided as Supporting Information is already fully in English. The non-English content identified corresponds to the original ethics approval document issued in Japanese. As required during the initial submission, we have provided an official English-language certificate of approval issued by our institution as Supporting Information. This document represents the formal and certified version for international use.

Therefore, we have retained the official English version and removed the Japanese version to comply with the journal’s language requirements.

6. Data anonymization and availability

Response:

We have revised the Supporting Information dataset to ensure full anonymization in accordance with PLOS data sharing policies. Specifically, exact age values have been replaced with categorized age groups to eliminate any potential risk of participant identification.

All other potentially identifying variables had already been removed in the original submission. The revised dataset therefore contains no direct or indirect identifiers and fully complies with participant privacy protection requirements.

In addition, we have confirmed with our institutional ethics committee that the anonymized dataset can be shared publicly in accordance with applicable regulations.

Response to the comments from the reviewer 1

Comment 1. The definition of “healthy individuals” should be clarified more clearly.

The manuscript repeatedly describes the validation cohort as “healthy individuals.” However, the actual cohort includes living donor candidates, patients undergoing elective laparoscopic cholecystectomy, and patients who underwent contrast-enhanced CT for acute abdomen, trauma, urologic disease, gonadal disease, and other conditions. In the current form, it is not clear how “healthy” was defined in this study and what exclusion criteria were applied. Please explain in more detail how subjects with possible liver dysfunction, chronic liver disease, nutritional problems, or other conditions that may affect liver volume were assessed and excluded. This point is important because the reliability of the reference TLV depends on the appropriateness of the study population.

Response:

We thank the reviewer for this important and insightful comment.

We agree that the term “healthy individuals” may not have been sufficiently defined in the original manuscript and could lead to ambiguity. We have therefore revised the manuscript to clarify the definition of the validation cohort and the inclusion/exclusion criteria in greater detail.

Specifically, the cohort consisted of three groups:

1 Living donor candidates, who underwent extensive clinical, biochemical, and imaging evaluation and had no evidence of liver disease, including steatosis. These individuals can be considered physiologically normal by definition.

2 Adult patients undergoing elective laparoscopic cholecystectomy for benign gallbladder disease. These patients were included consecutively to minimize selection bias. Although minor laboratory abnormalities may have been present in some cases, individuals with known chronic liver disease, malignancy, or conditions affecting liver morphology were excluded. We intentionally adopted this consecutive inclusion strategy to enhance generalizability, accepting a trade-off between strict physiological normality and real-world applicability.

3 Pediatric patients who underwent contrast-enhanced CT for conditions unrelated to hepatobiliary or malignant disease. Cases with any known liver pathology were excluded.

Accordingly, we have revised the wording in the manuscript to avoid the potentially misleading term “healthy individuals” and instead describe the cohort as a “clinically normal population without known liver disease.”

We believe this approach provides a more realistic and generalizable reference for CT-measured TLV, while maintaining sufficient biological validity for the purpose of external validation.

The revised parts are as follows:

Page 2, lines 13-14 (new), page 6, lines 17-18 (new), and page 20, line 6 (new)

individuals without known liver disease

Page 7, line 22-page 8, line 5 (new)

Individuals with known chronic liver disease, malignancy, or structural liver abnormalities were excluded. Living donor candidates were considered physiologically normal based on comprehensive clinical evaluation, including laboratory testing and imaging. For non-donor subjects, we adopted a consecutive inclusion strategy to minimize selection bias, accepting a trade-off between strict physiological normality and generalizability.

Comment 2. More methodological detail is needed for CT volumetry.

The manuscript states that TLV was measured using contrast-enhanced CT and three-dimensional image analysis software, but the actual measurement procedure is not sufficiently described. Please clarify whether liver segmentation was manual, semi-automatic, or automatic, whether major intrahepatic vessels were included or excluded, and how focal hepatic lesions or other factors potentially affecting liver volume were handled. Since this study is essentially a validation study, the reliability of the CT-based reference standard should be described more clearly.

Response:

We have revised the Methods section to provide additional detail on the measurement procedure. TLV was measured using contrast-enhanced CT images with 1-mm slice thickness and three-dimensional image analysis software (Synapse VINCENT; Fujifilm Corporation, Tokyo, Japan). Liver segmentation was performed using a semi-automated approach, in which hepatic contours and intrahepatic vascular structures were initially detected automatically and subsequently reviewed and, if necessary, manually adjusted by experienced operators.

Major intrahepatic vessels were excluded from the volume calculation, and focal hepatic lesions, all of which were benign in this cohort, were also excluded. All segmentations were visually confirmed to ensure accuracy.

Although the software used in this study allows detailed segmental volumetry, the present analysis focused exclusively on total liver volume (TLV), and segmental volume measurements were not used. This point has been clarified in the manuscript to avoid potential confusion.

We believe that these additions clarify the reliability of CT-based volumetry as the reference standard in the present validation study.

The revision is as follows:

Page 8, lines 8-18 (new)

TLV was measured using contrast-enhanced CT images with 1-mm slice thickness and three-dimensional image analysis software (Synapse VINCENT; Fujifilm Corporation, Tokyo, Japan). Liver segmentation was performed using a semi-automated approach, in which hepatic contours and intrahepatic vascular structures were initially detected automatically and subsequently reviewed and, if necessary, manually adjusted by experienced operators. Major intrahepatic vessels were excluded from volume calculation, and focal hepatic lesions, all of which were benign in this cohort, were also excluded. Although the software allows detailed segmental volumetry, the present study focused exclusively on TLV, and segmental volume measurements were not used in the analysis.

Comment 3: The discussion on race or population differences should be shortened and toned down.

The discussion about racial explanation is interesting, but it is somewhat too long and too assertive compared with the main message of this study. Since the present analysis was performed in a single Japanese cohort, the manuscript should be more careful when making broader statements about the absence of population-related differences. A shorter and more focused discussion would improve the balance of the paper.

Response:

We thank the reviewer for this important and thoughtful comment.

We agree that the original discussion on population and racial differences was overly long and somewhat assertive relative to the main focus of the present study. Accordingly, we have substantially shortened and revised this section to improve balance and clarity.

In the revised manuscript, we have removed broader statements regarding human genetic diversity and focused the discussion on evidence directly relevant to liver volume. Specifically, we retained (i) the autopsy-based study demonstrating no difference in liver-to-body weight ratios between African and European individuals, (ii) our own validation results showing that unbiased equations were not confined to a single derivation population, and (iii) the meta-analysis by Johnson et al. reporting no difference in TLV normalized for body surface area between Japanese and European individuals.

At the same time, we have moderated the tone of the discussion and avoided overly definitive statements. The revised text now emphasizes that discrepancies among SLV equations are more plausibly explained by differences in derivation age range, allometric scaling, and age-related hepatic involution, rather than attributing them primarily to population origin.

We believe that these revisions address the reviewer’s concern while preserving the methodological implications of our findings.

The revised corresponding part of Discussion section is as follows:

Page 18, line 4-page 19, line 13 (new)

In Western clinical practice, particularly in extended liver resection, the equation proposed by Vauthey et al. has been widely adopted [6]. Discrepancies among SLV equations derived from Western and Asian, particularly Japanese, populations have often been attributed to population or racial differences, with the implication that equations derived from Japanese cohorts may underestimate TLV in Western populations [6,16,18]. However, such an interpretation may be premature if differences in derivation age range, allometric model structure, and age-related hepatic involution are not adequately considered.

A large autopsy-based study demonstrated that organ-to-body weight ratios, including that of the liver, do not differ between individuals of African (n = 149) and European (n = 172) descent [32]. Although this comparison does not directly include East Asian populations, this relationship is likely to extend to European and East Asian populations, given that genetic diversity is greater within African populations than between non-African populations as a result of the out-of-Africa bottleneck [33].

In addition, in the present study, validation was performed in a Japanese cohort, yet the SLV equations that demonstrated no substantial bias were not confined to any single derivation population. Among the unbiased equations, several were derived from European, Japanese, and other East Asian cohorts, indicating that model validity did not segregate by population origin.

Furthermore, this observation is consistent with the meta-analysis by Johnson et al., which reported no difference in TLV normalized for body surface area between Japanese and European individuals [10].

Taken together, these findings suggest that discrepancies among SLV equations are more plausibly explained by differences in derivation age range, allometric scaling, and age-related hepatic involution than by population origin alone.

Comment 4. There is an internal contradiction in the Data Availability Statement.

In the manuscript, the Data Availability Statement says that all relevant data are within the paper and its Supporting Information files. However, in the later Data availability section, the authors state that the raw data will be made available upon due request from the corresponding author. These two statements are not consistent and should be corrected.

Response:

We agree that the original manuscript contained contradictory statements regarding data availability. To resolve this issue, we removed the statement indicating that raw data would be made available upon request from the corresponding author.

The revised manuscript now consistently states that all relevant data are available within the paper and its Supporting Information files, in accordance with PLOS ONE data sharing policies.

Minor comment: Some expressions are too rhetorical for a scientific paper. For example, the “Tower of Babel” expression in the Introduction, the Einstein/Newton analogy in the Conclusion, and the quotation from T. S. Eliot may be too literary for this type of article. A more neutral style would be preferable.

Response:

We thank the reviewer for this thoughtful suggestion.

We agree that some expressions in the original manuscript were more conceptual and metaphorical than is typical for a scientific article. However, our intention was to communicate complex methodological relationships in a manner that would be intuitively understandable to readers, particularly the idea that previously proposed SLV equations should not necessarily be regarded as mutually exclusive or competing constructs.

Accordingly, we revised and moderated several expressions to improve the scientific tone of the manuscript. Specifically, the quotation from T. S. Eliot was removed, and the “Tower of Babel” expression was slightly softened. However, the analogy between classical and more general physical theories was retained in a moderated form because it conveys the central conceptual message of the study, namely that broader physiological frameworks may incorporate previously proposed equations as locally valid approximations rather than replacing them entirely.

We hope that these revisions improve the overall balance and readability of the manuscript while preserving its conceptual clarity. We would, of course, be happy to make further modifications should the editor consider additional changes necessary.

The revisions are as follows:

Page 5, line 1 (new).

a situation that has created what may be described as a “Tower of Babel” in the SLV literature.

Page 21, line 6 (new).

Every robust scientific model ideally incorporates previous frameworks as locally valid approximations within a broader and more general theory.

Page 20, lines 11-14 (old) deleted

In essence, this study returns to the foundations of SLV estimation with a broader and more integrative perspective. As T. S. Eliot wrote, “the end of all our exploring will be to arrive where we started and know the place for the first time.”

Page 22, lines 2-3 (new).

and instead reinterpreting existing models within an age-aware physiological framework.

Response to the comments from the reviewer 2

Comment 1. It is mentioned in the conclusion (lines 9-10, page 10) that "incorporation of age adjustment into clinical practice may improve transplantation planning." You can add 1-2 sentences here to specify how to do this. For example, would it be recommended to develop a new formula that includes an age factor, or would it be recommended to multiply directly by an age-corrected factor (e.g., every 5 years afte

Attachment Submitted filename: Response yp Reviewers.docx

# Peer review: Decision Letter 1

**DOI:** 10.1371/journal.pone.0351983.r003

**Authors:** Yun-Wen Zheng

3 Jun 2026

Age-range–matched external validation of standard liver volume equations: methodological re-evaluation of 13 regression models

PONE-D-26-09031R1

Dear Dr. Imamura,

We’re pleased to inform you that your manuscript has been judged scientifically suitable for publication and will be formally accepted for publication once it meets all outstanding technical requirements.

Within one week, you’ll receive an e-mail detailing the required amendments. When these have been addressed, you’ll receive a formal acceptance letter and your manuscript will be scheduled for publication.

An invoice will be generated when your article is formally accepted. Please note, if your institution has a publishing partnership with PLOS and your article meets the relevant criteria, all or part of your publication costs will be covered. Please make sure your user information is up-to-date by logging into Editorial Manager at Editorial Manager® and clicking the ‘Update My Information' link at the top of the page. For questions related to billing, please contact billing support.

If your institution or institutions have a press office, please notify them about your upcoming paper to help maximize its impact. If they’ll be preparing press materials, please inform our press team as soon as possible -- no later than 48 hours after receiving the formal acceptance. Your manuscript will remain under strict press embargo until 2 pm Eastern Time on the date of publication. For more information, please contact onepress@plos.org.

Kind regards,

Yun-Wen Zheng

Academic Editor

PLOS One

Additional Editor Comments (optional):

Reviewers' comments:

Reviewer's Responses to Questions

**Comments to the Author**

1. If the authors have adequately addressed your comments raised in a previous round of review and you feel that this manuscript is now acceptable for publication, you may indicate that here to bypass the “Comments to the Author” section, enter your conflict of interest statement in the “Confidential to Editor” section, and submit your "Accept" recommendation.

Reviewer #1: All comments have been addressed

Reviewer #2: All comments have been addressed

**********

2. Is the manuscript technically sound, and do the data support the conclusions?

The manuscript must describe a technically sound piece of scientific research with data that supports the conclusions. Experiments must have been conducted rigorously, with appropriate controls, replication, and sample sizes. The conclusions must be drawn appropriately based on the data presented.

Reviewer #1: Yes

Reviewer #2: Yes

**********

3. Has the statistical analysis been performed appropriately and rigorously?

Reviewer #1: Yes

Reviewer #2: Yes

**********

4. Have the authors made all data underlying the findings in their manuscript fully available?

The PLOS Data policy requires authors to make all data underlying the findings described in their manuscript fully available without restriction, with rare exception (please refer to the Data Availability Statement in the manuscript PDF file). The data should be provided as part of the manuscript or its supporting information, or deposited to a public repository. For example, in addition to summary statistics, the data points behind means, medians and variance measures should be available. If there are restrictions on publicly sharing data—e.g. participant privacy or use of data from a third party—those must be specified.

Reviewer #1: Yes

Reviewer #2: Yes

**********

5. Is the manuscript presented in an intelligible fashion and written in standard English?

PLOS ONE does not copyedit accepted manuscripts, so the language in submitted articles must be clear, correct, and unambiguous. Any typographical or grammatical errors should be corrected at revision, so please note any specific errors here.

Reviewer #1: Yes

Reviewer #2: Yes

**********

6. Review Comments to the Author

Please use the space provided to explain your answers to the questions above. You may also include additional comments for the author, including concerns about dual publication, research ethics, or publication ethics. (Please upload your review as an attachment if it exceeds 20,000 characters)

Reviewer #1: I thank the authors for their careful revision and detailed point-by-point responses. The revised manuscript has substantially improved and now largely addresses the concerns raised in the previous review.

The authors have clarified the study population by replacing the potentially misleading term "healthy individuals" with a more appropriate description, namely individuals without known liver disease, and added detail on the exclusion criteria. The methodological description of CT volumetry has also been improved: liver segmentation was performed by a semi-automated method with manual correction when necessary, and major intrahepatic vessels and focal benign lesions were excluded. These additions make the reference standard more transparent and acceptable for a validation study.

The discussion of race or population differences has been shortened and moderated. Although some statements still remain somewhat broad, it is now more balanced and clearly focused on the main message, that differences in derivation age range and age-related hepatic involution are important sources of disagreement among SLV equations. The previous inconsistency in the Data Availability Statement has been corrected, and the supporting information was revised to improve anonymization.

The comments from Reviewer 2 have also been generally addressed, including a practical age-adjustment example after 50 years and the clinical implications of age-related liver volume reduction.

Overall, the revised manuscript satisfies the major PLOS ONE criteria for publication, and I believe it is suitable for publication.

Reviewer #2: (No Response)

**********

7. PLOS authors have the option to publish the peer review history of their article (what does this mean?). If published, this will include your full peer review and any attached files.

If you choose “no”, your identity will remain anonymous but your review may still be made public.

**Do you want your identity to be public for this peer review?**  For information about this choice, including consent withdrawal, please see our Privacy Policy.

Reviewer #1: No

Reviewer #2: No

**********

# Editor report: Acceptance letter

**DOI:** 10.1371/journal.pone.0351983.r004

**Authors:** Yun-Wen Zheng

PONE-D-26-09031R1

PLOS One

Dear Dr. Imamura,

I'm pleased to inform you that your manuscript has been deemed suitable for publication in PLOS One. Congratulations! Your manuscript is now being handed over to our production team.

At this stage, our production department will prepare your paper for publication. This includes ensuring the following:

* All references, tables, and figures are properly cited

* All relevant supporting information is included in the manuscript submission,

* There are no issues that prevent the paper from being properly typeset

You will receive further instructions from the production team, including instructions on how to review your proof when it is ready. Please keep in mind that we are working through a large volume of accepted articles, so please give us a few days to review your paper and let you know the next and final steps.

Lastly, if your institution or institutions have a press office, please let them know about your upcoming paper now to help maximize its impact. If they'll be preparing press materials, please inform our press team within the next 48 hours. Your manuscript will remain under strict press embargo until 2 pm Eastern Time on the date of publication. For more information, please contact onepress@plos.org.

You will receive an invoice from PLOS for your publication fee after your manuscript has reached the completed accept phase. If you receive an email requesting payment before acceptance or for any other service, this may be a phishing scheme. Learn how to identify phishing emails and protect your accounts at https://explore.plos.org/phishing.

If we can help with anything else, please email us at customercare@plos.org.

Thank you for submitting your work to PLOS ONE and supporting open access.

Kind regards,

PLOS ONE Editorial Office Staff

on behalf of

Dr. Yun-Wen Zheng

Academic Editor

PLOS One
